# Fungal and Bacterial Diversity Patterns of Two Diversity Levels Retrieved From a Late Decaying *Fagus sylvatica* Under Two Temperature Regimes

**DOI:** 10.3389/fmicb.2020.548793

**Published:** 2021-01-11

**Authors:** Sarah Muszynski, Florian Maurer, Sina Henjes, Marcus A. Horn, Matthias Noll

**Affiliations:** ^1^Department of Applied Science, Institute of Bioanalysis, University of Coburg, Coburg, Germany; ^2^Institute of Microbiology, Leibniz University of Hannover, Hanover, Germany

**Keywords:** microbial network analysis, bacterial and fungal community composition, *Fagus sylvatica*, fluctuating temperature regime, dead wood decomposition, insurance hypothesis

## Abstract

Environmental fluctuations are a common occurrence in an ecosystem, which have an impact on organismic diversity and associated ecosystem services. The aim of this study was to investigate how a natural and a species richness-reduced wood decaying community diversity were capable of decomposing *Fagus sylvatica* dead wood under a constant and a fluctuating temperature regime. Therefore, microcosms with both diversity levels (natural and species richness-reduced) were prepared and incubated for 8 weeks under both temperature regimes. Relative wood mass loss, wood pH, carbon dioxide, and methane emissions, as well as fungal and bacterial community compositions in terms of Simpson‘s diversity, richness and evenness were investigated. Community interaction patterns and co-occurrence networks were calculated. Community composition was affected by temperature regime and natural diversity caused significantly higher mass loss than richness-reduced diversity. In contrast, richness-reduced diversity increased wood pH. The bacterial community composition was less affected by richness reduction and temperature regimes than the fungal community composition. Microbial interaction patterns showed more mutual exclusions in richness-reduced compared to natural diversity as the reduction mainly reduced abundant fungal species and disintegrated previous interaction patterns. Microbial communities reassembled in richness-reduced diversity with a focus on nitrate reducing and dinitrogen-fixing bacteria as connectors in the network, indicating their high relevance to reestablish ecosystem functions. Therefore, a stochastic richness reduction was followed by functional trait based reassembly to recover previous ecosystem productivity.

## Introduction

An ecosystem is a complex network and commonly experiences environmental fluctuations (Levin, 1998), which have a high impact on ecosystem functions and its linked biodiversity parameters such as species richness, dispersal rate, and community composition (Loreau et al., 2003; Norberg, 2004). The degree to which an ecosystem function can resist or recover rapidly from environmental perturbations was defined as recovery, which can also affect the productivity of an ecosystem (Oliver et al., 2015). Yachi and Loreau (1999) theoretically modeled for an ecosystem under a fluctuating environment that recovery and productivity are increasing with increasing species richness (Yachi and Loreau, 1999) and named this model insurance hypothesis. Several non-experimental studies investigated this hypothesis by testing the effect of climate data (DeClerck et al., 2006) and of modeled community compositions (Ives et al., 2000). In addition, this hypothesis was experimentally tested in aquatic environments (Leary and Petchey, 2009) and forest environments (Toljander et al., 2006). Results of the majority of these studies indicate that increasing species richness stabilized ecosystem functions (Ives et al., 2000; Toljander et al., 2006; Leary and Petchey, 2009).

Dead wood decay has an ecological impact in forest ecosystems and can last few years up to many decades. Temperature strongly influences the dead wood decay (A’Bear et al., 2014), and is a major driver for microbial activity and the linked ecosystem services (Biederbeck and Campbell, 1973; Pietikäinen et al., 2005; Conrad et al., 2009). Aerobic dead wood decay is primarily driven by white rot, brown rot, soft rot and mold fungi (Leonhardt et al., 2019). Commonly, these fungi are associated with a large diversity of bacterial communities that are metabolizing wood components directly or the metabolites from fungal dead wood decay (Ausmus, 1977; Hoppe et al., 2014; Moll et al., 2018; Lasota et al., 2019). Both fungal and bacterial community composition, activity and organismal interactions are shaped by dead wood tree species (Prewitt et al., 2014), moisture (Hu et al., 2017), dead wood C quality (Hu et al., 2017), dead wood density (Kielak et al., 2016), forest stand type (Kahl et al., 2017), and exposure time of dead wood (Kahl et al., 2017). At two controlled temperature environments, Toljander and colleagues (2006) found that artificial community compositions of boreal brown rot and white rot fungi were not linked to species richness but to niche differentiation (Toljander et al., 2006). Wood decaying communities are composed of several bacterial and fungal species, which are part of community interaction networks (Hoppe et al., 2014; Kielak et al., 2016; Hu et al., 2017). The community composition and its functional diversity change along dead wood decay succession, and each microbial community composition have its own productivity constraints (Valentín et al., 2014). For instance, a late spruce dead wood decaying community was more resistant to the loss of diversity by species richness reduction than an early stage community (Valentín et al., 2014). However, how environmental fluctuations as temperature and a reduction of a natural beech decaying microbial community richness affects wood decomposition was to best of our knowledge so far not addressed.

The aim of this study was to investigate (i) that increased species richness buffers decomposition processes against environmental fluctuations; (ii) the combined effect of temperature regime and species richness on microbial community development; and (iii) the combined effect of temperature regime and species richness on community co-occurrence networks. Therefore, a natural late decaying microbial community, was retrieved from dead wood logs of *Fagus sylvatica*, which was exposed for 8 years in three forest sites. The natural community was subsequently 10 times diluted by similar sterilized wood samples (richness-reduced diversity, R) and compared with the undiluted community (natural diversity, N). Richness-reduced and natural community were incubated under oxic conditions for 8 weeks under a constant and a fluctuating temperature regime.

## Materials and Methods

### Sampling Area and Sampling Campaign

The sampling area is located in the Hainich National Park in Central Germany (N51.08, E 10.43) in Thuringia which is one of three experimental areas of the biodiversity exploratories^1^ (Fischer et al., 2010). The samples were taken in summer 2017 from dead wood *F. sylvatica* logs, which were exposed since 2009, in three different experimental plots (HEW 3, HEW 5, and HEW 12) (Kahl et al., 2017). Thirty nine dead wood chip samples (3 plots × 13 replicate drillings per log) were sampled by drilling holes (Ø 40 mm, 100 mm into wood, 45° angle) with a cordless drill (Makita DHP 451, Brussels, Belgium) equipped with a Forstner bit as explained earlier Hoppe et al. (2014). Wood chips were subsequently packed in autoclave bags and stored at 5°C during the rest of the fieldwork.

### Experimental Design

All wood chips were pooled and one part remained untreated while another part were gamma-sterilized by 30 kGy by Synergy Health Radeberg GmbH (Radeberg, Germany). Thereafter, environmental and gamma-sterilized wood chips were separately milled under sterile conditions with a coffee grinder (3 min, max. power) to retrieve a similar homogeneous wood matrix. Three diversity levels were prepared thereafter, (i) natural diversity [N: 10 g (dry weight) untreated wood chips], (ii) richness-reduced diversity [R: 9 g (dry weight) gamma-sterilized wood chips and 1 g untreated wood chips], and (iii) sterile diversity [S: 10 g (dry weight) of gamma-sterilized wood chips] in 10 replicates for each incubation time point (0, 1, 2, 3, 4, 5, 6, 7, and 8 weeks). Each of the wood chips were added to a 100 ml glass vial and wood moisture was adjusted to 50% (vol/wt) according to Jakobs-Schönwandt et al. (2010) and thereafter airtight closed. All diversity levels were randomly split in two temperature regimes. The constant temperature regime had a constant temperature at 10°C (c), while the fluctuating temperature regime (f) changed each 7 days from 3 to 13°C to 18 to 5°C and 3 to 13°C to 18 to 5°C with a temperature ramping of 1°C per minute, and a mean temperature of 10°C. After each week of incubation five replicates of each diversity level (n_*n*_ = 5, n_*R*_ = 5, n_*S*_ = 5) and of both temperature regimes (n_*c*_ = 5, n_*f*_ = 5) were used to determine relative wood mass loss and wood pH. Initial wood mass was subtracted from wood mass loss after incubation of each diversity level and named relative wood mass loss. The wood pH was measured with an extraction method in triplicates according to Humar et al. (2001). All wood chips of all diversity levels were milled with a coffee grinder to fine wood debris and 50 ml of boiling water was added. The extracts were steeped for 5 min and subsequently rapidly cooled in an ice bath for 30 min to room temperature. Afterward the pH of the wood extracts were measured.

### Gas Measurements

In total, 90 gas samples were taken (each diversity level in both temperature regimes after 1, 4 and 8 weeks of incubation in five replicates) with a 10 ml gas tight needle (Hamilton, Reno, NV; United States) and transferred into 3 ml gastight vails (Exetainer^®^, Labco, United Kingdom) and stored at room temperature. To detect concentration changes in carbon dioxide, methane and oxygen, gases were quantified using an Agilent 7,890 gas chromatograph (Agilent Technologies Inc., Santa Clara, CA, United States) equipped with a ShinCarbon ST 80/100 column (2 m, 0.5 mm ID, Restek Corporation, Bellafonte, PA, United States) and a pulsed-discharge helium ionization detector. Oxygen and oxygen-dinitrogen ratios were used as indicator for aerated incubations to monitor aerobic wood decay.

### Bacterial and Fungal Community Composition

In total 255 samples (2 diversity levels [N, R] × 2 temperature regimes [c, f] of 5 replicates × 8 time points [1, 2, 3, 4, 5, 6, 7,8 weeks of incubation]) + (2 diversity levels [N, R] of 5 replicates [start of experiment]) + (sterilized wood chips [S] × 2 temperature regimes of 5 replicates × 8 time points) + (1 start of experiment of 5 replicates) were pulverized with liquid N_2_ in a vibration mill MM 400 (Retsch GmbH, Haan, Germany) for 3 min at 30.0 Hz (milling cup: 50 ml, steal ball Ø 5 cm). Genomic DNA were extracted from 200 mg pulverized wood by using the Quick-DNA Fecal/Soil Microbe Miniprep Kit (Zymo Research, Freiburg, Germany) as outlined in the manufacturer’s protocol. Presence and purity of the extracted nucleic acids were determined by measuring the optical density at 260 nm with a spectrophotometer Multiskan^TM^ Go (Thermal Fisher Scientific GmbH, Schwerte, Germany).

Bacterial 16S rRNA gene was amplified using the 341F (5’CCTACGGGNGGCWGCAG’3) and 785R (5’GACTACHVGGGTATCTAATCC’3) primer pair (Thijs et al., 2017), whereas the fungal ribosomal internal transcribed spacer 2 region (ITS2) was amplified using the FITS7 (5′GTGARTCATCGAATCTTTG ′3) and ITS4 (5′TCCTCCGCTTATTGATATGC ′3) (Tedersoo et al., 2015) primer pair. PCR products were used for subsequent 300 bp paired-end sequencing with Illumina Miseq V3 System (San Diego, CA, United States), which was carried out by LGC Genomics (LGC Genomics GmbH, Berlin, Germany). Sequence raw data were de-multiplexed by using the Illumina bcl2fastq 2.17.1.14 software (bcl2fastq2 Converstion Software v2.20) and were subsequently sorted by reads of amplicon inline barcodes. Barcode sequences, adapters and primers were clipped from the sequence and forward and reverse reads were combined by using BBMerge 34.48 (Bushnell et al., 2017). 16S rRNA gene and fungal ITS2 region sequences were pre-processed and operational taxonomic units (OTUs) were picked from the amplicons with Mothur 1.35.1 (Schloss et al., 2009). Sequences with ambiguous bases, with homopolymer stretches and with an average quality score below 33 were removed (Whelan et al., 2019). Short products were removed and chimeras were eliminated with the UCHIME algorithm (Edgar et al., 2011). For 16S rRNA gene sequences, an alignment against the 16S Mothur-Silva SEED r119 reference (Schloss et al., 2009) was performed and OTUs were picked by clustering at the 97% identity level and taxonomical classified against the Silva reference classification (Buettner and Noll, 2018). Sequences from other domains of life were removed. ITS2 region sequences were CD-HIT-EST clustered at 97% identity level to the most abundant sequence and taxonomical classified against the UNITE version 6 reference database. In addition, OTUs were assigned to ecological functional groups of bacteria and fungi using functional annotation of prokaryotic taxa FAPROTAX (Louca et al., 2016) and FUNGUILD (Nguyen et al., 2016), respectively.

### Statistics and Network Analysis

The relative abundance of bacterial and fungal community OTU composition was calculated as explained by Noll et al. (2005). Briefly, the sequencing depth was normalized to the lowest sequence read amount of 31,405 bacterial and 1,646 fungal sequence to 903 bacterial and 384 fungal by summarizing identical OTU’s. Thereafter, the percentage OTU abundance (A*p*) of sample was calculated as A*p* = *n*_*i*_ × 100/N in which *n*_*i*_ represents the sequence reads of one distinct bacterial and fungal OTU, and N is the sum of all bacterial and fungal sequence reads in one sample, respectively. To reduce data noise, only A*p*-values ≥ 1% for both the bacterial and fungal OTU data set were considered for further analyses. After exclusion of minor OTUs (< 1%), the A*p*-values were recalculated. OTUs of the same taxonomic classification were pooled and OTUs, which were found only in the sterile diversity (see Supplementary Table S1) but not in the natural or richness-reduced diversity were removed.

Descriptive ecological community data analysis was carried out in the R statistical environment (RStudio Team, 2015; R Core Team, 2019) and the corresponding R package “vegan” (ver. 2.5-5) (Oksanen et al., 2019). Constrained correspondence analysis (CCA) was calculated to correlate bacterial and fungal relative OTU abundances, respectively with relative wood mass loss, wood pH, diversity level (N, R), temperature regime (f, c), incubation time, carbon dioxide and methane concentration. The significant effect of each variable on bacterial and fungal OTU abundances were tested by the anova.cca function in R package vegan (ver. 2.5-5) with 9999 replicate runs, and significance level was set as *p* = 0.05. In addition, beta diversity was calculated by a Bray Curtis dissimilarity matrix and analyzed with a non-metric multidimensional scaling (NMDS). The Simpson‘s diversity index, richness and evenness were calculated for both the bacterial and fungal OTU abundances and their effect on diversity level, temperature regimes, relative wood mass loss, wood pH and incubation time was compared by a two way ANOVA in Rstudio (*p* ≤ 0.05). Normal distribution was assumed (*N* = 255) and homogeneity of variances was tested, if no homogeneity of variances was given the robust oneway.test instead of an ANOVA was calculated. The effect of both temperature regimes were calculated by an one-way ANOVA for relative wood mass loss, wood pH, methane and carbon dioxide concentration for each diversity level separately.

Network analyses of bacterial and fungal OTUs of natural and richness-reduced community were carried out in the software Cytoscape (ver. 3.7.2) (Shannon et al., 2003) and the add-in CoNet (Faust and Raes, 2016). The networks were computed for each incubation time point to analyze temporal shifts in the OTU co-occurrence patterns. First, the relative OTU abundance matrix of both bacterial and fungal OTUs of natural and richness-reduced diversity, respectively, were loaded without pre-processing in CoNet, including the respective taxonomic affiliations and parameters (wood mass loss, wood pH and temperature regimes). Preliminary networks were computed with the Spearman- and Kendall correlations, the Bray Curtis dissimilarity and the Hellinger distance as these methods were robust and non-sensitive by high frequency of zero values (Faust and Raes, 2016). The top and bottom edge numbers were set to 2,500 and force intersection was enabled (Faust and Raes, 2016). Only co-occurrence patterns of bacterial and fungal OTUs were included, that were supported by minimum of three out of four correlation methods. Significant co-occurrence patterns (*p* = 0.05) for all network edges were tested by a permutation test with 100 iterations and a matrix row-based resampling method (Faust and Raes, 2016). Thereafter, edge specific confidence interval were tested by a 100 iteration bootstrapping method (Faust and Raes, 2016). Finally a multiple test correction (Benjamini and Hochberg, 1995) and an edge specific *p*-value merge method (Brown, 1975) were carried out and the final networks were calculated. Each node and edge of the final calculated network were assigned to one specific cluster by using GLay community algorithm (Su et al., 2010). Afterward, for each prior assigned cluster intramodularity (Zi) and intermodularity (Pi) were calculated as outlined previously (Guimerà and Amaral, 2005). Afterward for each OTU an assignment to network roles was carried out as described by Olesen et al. (2007), where OTU with Zi > 2.5 and Pi < 0.62 were characterized as module hubs, Zi > 2.5 and Pi > 0.62 as network hubs, Zi < 2.5 and Pi < 0.62 as peripherals and Zi < 2.5 and Pi > 0.62 as connectors. All figures were plotted with the R package ggplot2 (version 3.3.2) in R statistical environment.

## Results

### Wood Degradation Under Two Temperature Regimes for Two Diversity Levels

**FIGURE 1:**
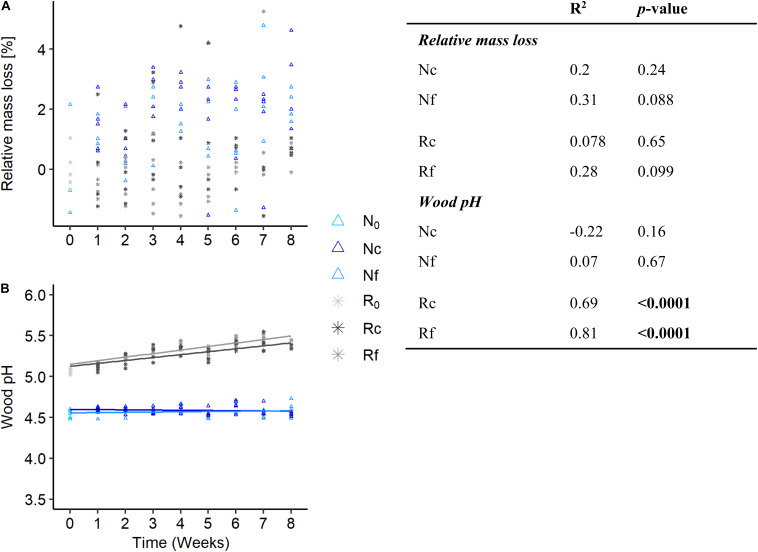
Relative wood mass loss **(A)** and wood pH **(B)** from *Fagus sylvatica* dead wood chips under two temperature regimes (fluctuating, f and constant, c) and two diversity levels (natural, N and richness-reduced, R) over 8 weeks of incubation. Relative wood mass loss was defined as percentage change from the initial wood mass (N_0_ and R_0_). Correlation coefficient was calculated for each diversity level (N, R) and temperature regime (f, c) and was indicated by a linear regression (see table to the figure). Significant correlation (*p* ≤ 0.05) is highlighted in bold. See figure legend for symbol of each diversity level and temperature regime.

**FIGURE 2:**
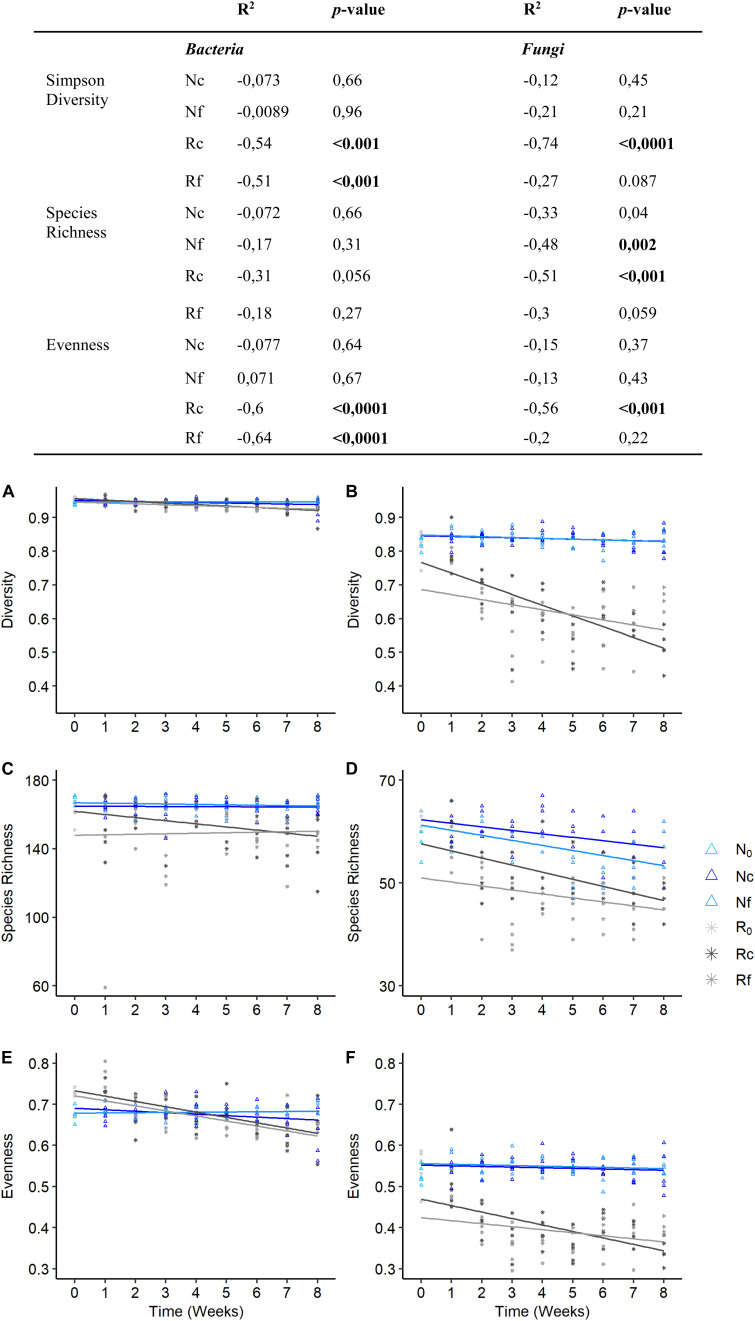
Simpson’s diversity, species richness and evenness for bacterial **(A,C,E)** and fungal **(B,D,F)** community structures of *Fagus sylvatica* dead wood chips under two temperature regimes (fluctuating, f and constant, c) and two diversity levels (natural, N and richness-reduced, R) over 8 weeks of incubation. Correlation coefficient of Simpsons diversity, species richness and evenness of two diversity levels and temperature regimes was indicated by a linear regression (see table to the figure). Significant correlation (*p* ≤ 0.05) is highlighted in bold.

The relative wood mass loss non-significantly increased in the fluctuating temperature regime compared to the constant temperature regime over time irrespectively of the diversity level (Figure 1A and Figure 2). Wood pH increased significantly over time in the richness-reduced diversity, while the pH in the natural diversity remained constant at 4.6 (Figure 1B). Moreover, the temperature regime significantly affected the relative wood mass loss, wood pH and the composition of bacterial and fungal diversity in the richness-reduced diversity treatment (Figure 2, Supplementary Figure S2, and Table 1). Carbon dioxide concentration increased significantly in the richness-reduced diversity for both temperature regimes over time, whereas methane concentrations as indicators for anaerobic metabolism decreased significantly over time only in the natural diversity (Supplementary Figure S3). No shifts in relative wood mass loss and wood pH were observed in the sterile diversity (Supplementary Figure S1 and Supplementary Table S1).

**TABLE 1:** The effect of temperature regime (fluctuating and constant) on relative wood mass loss, wood pH, bacterial and fungal simpson diverstiy, carbon dioxide and methane concentration of a richness-reduced (R) and natural (N) diversity level. Significance was determined by an one-way analysis of variance.

	*df*	Sum of square	Mean of square	*F*-value	*p*-value
**Richness-reduced diversity level (R)**					
Relative wood mass loss^*c*^	1	8.89	8.887	5.481	**0.0219**
Wood pH^*f*^	1	0.073	0.073	4.311	**0.041**
Bacterial community^*c*^	2	0.002	0.001	4.681	**0.012**
Fungal community^*f*^	2	0.1951	0.0975	9.839	**<0.005**
Methane^*f*^	1	0.164	0.164	0.618	0.438
Carbon dioxide	1	2227	2227	0.40	0.532
**Natural diversity level (N)**					
Relative wood mass loss^*c*^	1	0.0	0.0001	0.00	0.994
Wood pH^*c*^	1	0.0041	0.0041	1.366	0.246
Bacterial community	2	0.0002	0.00008	0.88	0.419
Fungal community	2	0.0434	0.0005	1.051	0.354
Methane^*c*^	1	0.098	0.098	2.278	0.143
Carbon dioxide	1	439188	439188	1.658	0.209

**Significance differences is highlighted in bold (p ≤ 0.05). df, degrees of freedom; ^*c*^, in constant temperature regime higher; ^*f*^, in fluctuating temperature regime higher.**

### Bacterial Community Composition Recovered Better Compared to Fungal Community Composition

The bacterial composition was dominated by members of the phyla Acidobacteria (subclass 1), Actinobacteria, Bacteriodetes, Firmicutes, Alpha-, Beta, and Gammaproteobacteria, while the fungal composition was mainly composed by members of the order Helotiales, Saccharomycetales, Sordariomycetes, Agaricomycetes, and the genera Herpotrichiellaceae and Trichocomaceae. Richness-reduced community composition lost disproportional (based on reduced relative sequence read abundances) members of the bacterial genera *Mucilaginibacter*, *Rhodanobacter*, *Nocardiodes*, *Pedobacter* and of the bacterial families Xanthomonadaceae, Burkholderiaceae, and Chitinophagaceae, and of the fungal genera *Sugiyamaella*, *Phialocephala*, *Sistotrema*, and *Phanerochaete*.

**FIGURE 3:**
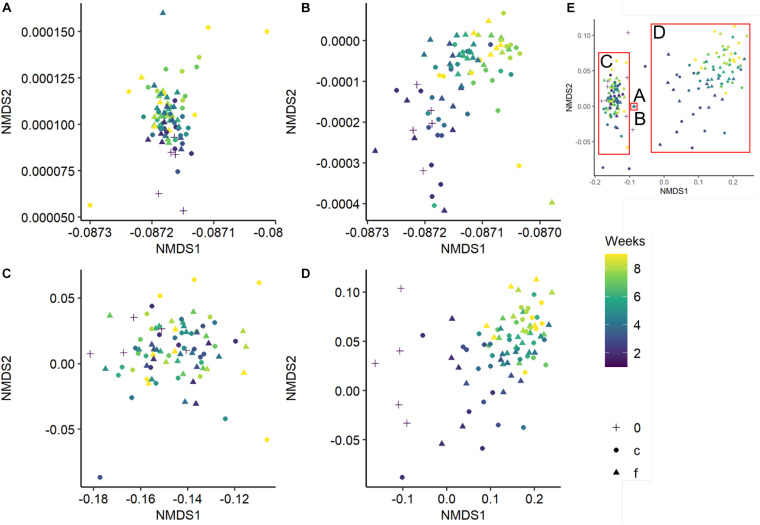
Non-metric multidimensional scaling (NMDS) of the bacterial natural **(A)**, bacterial richness-reduced **(B)**, fungal natural **(C)**, and fungal richness-reduced **(D)** community composition incubated in two temperature regimes (fluctuating, f ▲ and constant c 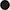). Overview of the NMDS sections **(A–D)** is comprised separately **(E)**. Community compositions were calculated of the relative OTU abundances after each week over an incubation period of 8 weeks (see color code of the symbols). Initial bacterial or fungal community composition is indicated as 0 (+). NMDS is based on a Bray-Curtis dissimilarity matrix. Further details can be found in the Supplementary Figure S4.

Bacterial and fungal community composition was significantly affected by wood pH, diversity level and incubation time, and fungal community composition was further influenced by temperature regime and relative wood mass loss (Table 2). Simpson‘s diversity index and evenness of the bacterial community were unaffected over incubation time and diversity level, while fungal and bacterial richness were significantly lower in the richness-reduced community composition compared to natural community composition (Figure 2C). In turn, Simpson‘s index, richness and evenness of the fungal community were significantly lower in the richness-reduced compared to the natural diversity (Figure 2). Moreover, all three indices of the richness-reduced diversity decreased in the constant temperature regime over time (Figure 2). Both the bacterial and the fungal community composition was dissimilar between richness-reduced and natural diversity (Figure 3). Moreover, the temporal shifts in the composition of the richness-reduced community were more pronounced compared to the natural community, while the effect on the temporal development on the composition of the temperature regimes were less important (Figure 3 and Supplementary Figure S4).

**TABLE 2:** The effect of relative wood mass loss, wood pH, diversity level (natural, N and richness-reduced, R), two temperature regimes (fluctuating and constant), incubation time (weeks), methane and carbon dioxide concentration on relative bacterial and fungal OTU abundance was determined by a constrained correspondence analysis.

	n	*df*	*X*^2^	*F*-value	*p*-value
**Bacteria**					
Relative wood mass loss	165	1	0.003978	2.01	0.0631
Wood pH	167	1	0.056714	28.66	**0.0001**
Diversity level (N, R)	167	1	0.011557	5.84	**0.0190**
Temperature (f, c)	167	2	0.006431	1.63	0.0790
Time	167	7	0.018267	1.32	**0.0287**
Methane	59	1	0.001941	0.35	0.9625
Carbon dioxide	59	1	0.003284	0.60	0.6931
**Fungi**					
Relative wood mass loss	165	1	0.01326	5.42	**0.0102**
Wood pH	167	1	0.27572	112.74	**0.0001**
Diversity level (N, R)	167	1	0.01235	5.05	**0.0130**
Temperature (f, c)	167	2	0.01845	3.77	**0.0014**
Time	167	7	0.02374	1.39	**0.0065**
Methane	59	1	0.00533	0.63	0.5676
Carbon dioxide	59	1	0.01132	1.33	0.2399

**Permutations of constrained correspondence analyses were calculated with 9,999 replicate runs and significance differences (p ≤ 0.05) is highlighted in bold. n, Sample number; df, Degrees of freedom; X^2^, Chi-Square.**

### Impact of Diversity Level on Microbial Networks and Co-occurrence Patterns

**FIGURE 4:**
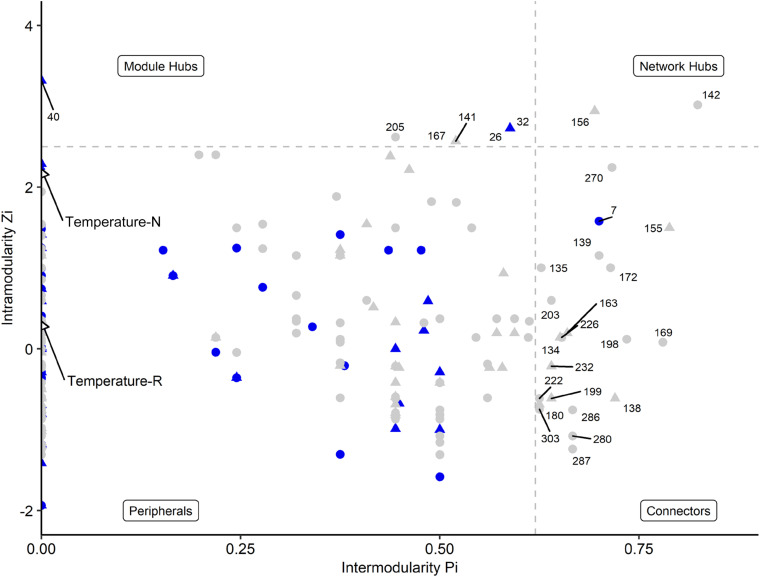
Network roles of each OTU of a microbial network analyses for natural (blue, N) and richness-reduced (gray, R) community composition after 8 weeks of incubation. Bacterial OTUs (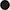), fungal OTUs (▲), and environmental parameters (◆) are denoted. Each OTU was categorized into network hubs, module hubs, connectors or peripherals according to Olesen et al. (2007) (see also Table 3 for additional information). Environmental parameters (see Table 2) were included and highlighted by arrows. Module hubs represent strong interactions inside a module, while connectors denote interactions outside a module. Network hubs have strong connections to both, inside and outside a module, while peripherals have neither of both interaction types.

Network analyses revealed different co-occurrence patterns of the natural and richness-reduced diversity over time where richness-reduced diversity underwent more shifts in co-occurrence patterns (Supplementary Table S2). After 8 weeks of incubation, connectors were more frequent in the richness-reduced (9.9% of all OTUs in the network after 8 weeks of incubation) compared to natural diversity (0.8%), but module hubs were equally distributed between both diversity levels (Figure 4 and Supplementary Table S2). Network hubs were only found in the richness-reduced diversity level. Functional traits of connector and module hub OTUs were more versatile in the richness-reduced compared to natural diversity (Table 3). Dinitrogen fixation and nitrate reduction were strongly associated in the richness-reduced bacterial diversity as well as white rot and wood saprotrophs in the fungal richness-reduced diversity.

**TABLE 3:** Taxonomic classification and functional trait of topological OTUs in a microbial network derived from the natural and richness-reduced diversity after 8 weeks of incubation.

#	Composition	Module	Taxon	Functional trait
**Bacterial community composition**				
7	Natural	Connector	*Enterobacteriaceae*	−
135	Reduced	Connector	*Chthoniobacterales*	−
139	Reduced	Connector	*Haloferula* sp.	Aerobic chemoheterotrophy
142	Reduced	Network hub	*Micropruina* sp.	Aerobic chemoheterotrophy; nitrate/nitrogen respiration; nitrate reduction
169	Reduced	Connector	*Taibaiella* sp.	−
172	Reduced	Connector	*Pigmentiphaga* sp.	−
198	Reduced	Connector	*Micrococcales*	−
203	Reduced	Connector	*Stenotrophomonas* sp.	Aerobic chemoheterotrophy, human pathogens, animal parasites/symbionts, nitrate/nitrogen respiration, nitrate reduction, atmospheric nitrogen fixation
205	Reduced	Module hub	*Opitutus* sp.	Fermentation, nitrate reduction
222	Reduced	Connector	*Verrucomicrobia*	−
226	Reduced	Connector	*Chryseobacterium* sp.	Aerobic chemoheterotrophy
270	Reduced	Connector	*Intrasporangiaceae*	−
280	Reduced	Connector	*Aquicella* sp.	Intracellular parasites
286	Reduced	Connector	*Defluviitaleaceae*	−
287	Reduced	Connector	*Bauldia* sp.	−
303	Reduced	Connector	*TM6*	−
**Fungal community composition**				
26	Natural	Module hub	*Pleosporales*	−
32	Natural	Module hub	*Lophodermium piceae*	Plant pathogen
40	Natural	Module hub	*Lophiostoma* sp.	Undefined saprotroph
134	Reduced	Connector	*Kretzschmaria deusta*	Undefined saprotroph
138	Reduced	Connector	*Chaetosphaeria* sp. *olrim419*	Endophyte, litter saprotroph, wood saprotroph
141	Reduced	Module hub	*Sordariomycetes*	−
155	Reduced	Connector	*Cryptococcus* sp.	Fungal parasite
156	Reduced	Network hub	*Polydesmia pruinosa*	Undefined saprotroph
163	Reduced	Connector	*Mycena haematopus*	Leaf saprotroph, plant pathogen, wood saprotroph
167	Reduced	Module hub	*Fibulorhizoctonia* sp.	White rot
180	Reduced	Connector	*Penicillium spinulosum*	Undefined saprotroph
199	Reduced	Connector	*Exophiala moniliae*	Animal pathogen, undefined saprotroph
232	Reduced	Connector	*Cladophialophora* sp. *olrim407*	Undefined saprotroph

*OTUs of the network were assigned to connectors, module hubs or network hubs according to Olesen et al. (2007) (see also Figure 4).*

**FIGURE 5:**
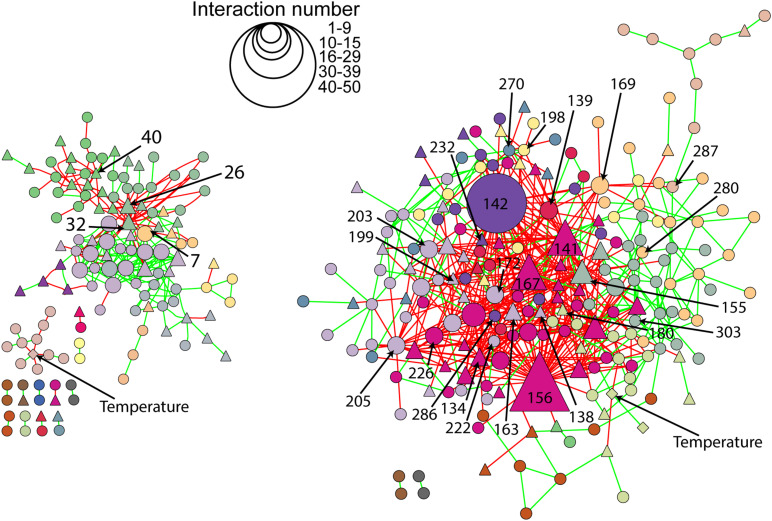
Network of natural (left) and richness-reduced (right) diversity after 8 weeks of incubation. Interaction types were represented as mutual exclusion (red lines) and co-presence (green lines) and symbol size indicate the number of interactions (see legend in figure). Bacterial OTUs (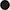), fungal OTUs (▲), and environmental parameters (◆) are denoted. Bacterial and fungal OTUs as well as environmental parameters of the same cluster are indicated in the same color. Additional information to the numbers of selected OTUs can be found in Figure 4 and Table 3.

The microbial co-occurrence patterns differed strongly between richness-reduced and natural diversity, and more OTUs were interactive in the richness-reduced diversity mainly with mutual exclusions (Figure 5). Very interactive OTUs in the richness-reduced diversity were *Polydesmia pruinosa* (network hub, 156) with 40 mutual exclusions as well as *Micropruina* sp. (network hub, 142). *Sordariomycetes* (module hub, 141) and *Fibulorhizoctonia* sp. (module hub, 167) had both 30 interactions including 28 mutual exclusions and two co-presences, while *Optitutus* sp. (module hub, 205) had 15 mutual exclusions and 15 co-presences. Interactive OTUs of the natural diversity were *Rhizomicrobium* sp. (peripheral, 22) with 17 co-presences as well as *Pleosporales* (module hub, 26) and *Lophodermium piceae* (module hub, 32) both with 17 mutual exclusions. In addition, *Lophiostoma* sp. (module hub, 40) had 8 mutual exclusions and one co-presence (Figure 5, Table 3 and Supplementary Table S3).

## Discussion

The main goal of the study was to clarify the effect of two temperature regimes on microbial dead wood decomposition under two diversity levels. A richness-reduced microbial diversity was more vulnerable to a fluctuating temperature regime, while a natural microbial diversity was better to recover in the same fluctuating temperature regime. Moreover, the bacterial community was more resistant to environmental diversity disturbances, than the fungal community. The further goal was to investigate microbial co-occurrence patterns in both diversity levels and the resulting patterns showed that an artificially richness-reduced community composition was strongly disturbed. Therefore, key players and its microbial network partners for wood decomposition were less-frequent present in the richness-reduced community.

Temperature regimes affected the richness-reduced diversity, which had an impact on wood decomposition and wood pH, while no impact for the natural diversity was observed (Figure 1 and Table 1). Therefore, loss of diversity caused also a loss of such ecosystem services (Figure 1), which is in line with similar studies (Hol et al., 2010; Valentín et al., 2014). We used a microbial community of beech dead wood of a natural forest as inoculum that previously reflected a high microbial diversity and dead wood decomposition activity (Kahl et al., 2017; Leonhardt et al., 2019). The dilution of such natural community obtained a richness-reduced community with 8 and 14% less of the total bacterial and fungal OTU richness, respectively (Figures 2C,D), which is in line with a previous study, where 11% of total fungal OTUs were received after similar dilution of late stage of spruce dead wood decay (Valentín et al., 2014). However, such dilutions affected mainly fungal OTU richness and evenness (Figure 2 and Supplementary Table S3), which may also affect associated activity patterns. Therefore, a richness-reduced diversity with a low evenness is more sensitive to environmental fluctuations, which has been shown previously (Griffiths et al., 2000) and is in accordance with the insurance hypotheses (Yachi and Loreau, 1999). Briefly, the insurance hypothesis predict that ecosystem productivity will be maintained close to its maximum value as long as species richness is high enough for the ecosystem to be redundant, but would decline abruptly when species richness is further reduced beyond this point (Yachi and Loreau, 1999). However, community composition (including richness, evenness) and its functional traits are more important for ecosystem productivity as both were indicators for functional redundancy and microbial recovery after environmental disturbance (Griffiths et al., 2000).

Similarly to this study, Valentín et al. (2014) observed a difference in carbon dioxide emission in both diversity levels in the late spruce decay stage, which is in line with higher carbon dioxide emissions of the richness-reduced diversity as these emissions were non-significantly increasing over time (Supplementary Figure S3). The carbon dioxide emissions was not correlated to mass loss in our study (Table 1) but in other studies (Valentín et al., 2014; Kahl et al., 2017). During wood decay, microbial activity is also linked to shifts in wood pH, which is mainly a decrease in wood pH due to fungal enzyme activities and non-enzymatic systems such as excretion of oxalic acid and the fenton reaction (Arantes et al., 2012). Wood pH increased up to 5.5 in the richness-reduced diversity, while the wood pH remained at 4.6 in the natural diversity (Figure 1B). However, wood degrading enzymes like laccase, endoglucanase, exoglucanase, and β-glucosidase have a pH optimum at 5.5 (Kluczek-Turpeinen et al., 2007; Ahmed et al., 2009) and fungi are capable of altering their environmental pH to their optimum (Kluczek-Turpeinen et al., 2007). Wood pH was significantly correlated to shifts in the bacterial and fungal community composition (Table 2), which can have a large influence on the interplay between fungal and bacterial communities (Rousk et al., 2008, 2010; Johnston et al., 2019). Moreover, the richness-reduced diversity significantly shifted wood pH up to 5.5 (Figure 1B), indicating that the microbial richness reduction caused a reorganization of microbial dominance in wood decay and thereby supporting activities of microorganisms which are capable to increase wood pH. In the same setting, an increase in relative sequence read abundances of bacterial and fungal OTUs such as *Scheffersomyces shehatae* (331), *Sphingomonas* sp. (257), *Terriglobus* sp. (237), *Granulicella* sp. (236), *Cadophora melinii* (176) (Supplementary Table S3) were observed in the richness-reduced diversity that are known as wood-inhabiting and wood decaying microorganisms (Eichorst et al., 2007; Lara et al., 2014; Yamada et al., 2014; Held and Blanchette, 2017; Worrall et al., 2018). However, wood pH conditions shape bacterial and fungal community composition in dead wood (Hiscox et al., 2016), and pH optimum of the majority of microorganisms in the richness-reduced community was in line with our pH conditions.

The bacterial community composition was less affected by the richness-reduced diversity level compared to fungal community composition (Supplementary Table S3), indicating that bacterial community composition was more resistant. Such findings were also reported in totally different ecosystems (Shade et al., 2011; Azarbad et al., 2015), indicating that bacterial community compositions undergo similar resistant strategies. Moreover, richness and evenness of bacterial OTUs were more similar between natural and richness-reduced diversity compared to fungal OTUs (Figure 2), underlining that fungal community composition was more sensitive to environmental and anthropogenic disturbances as shown previously (Tolkkinen et al., 2015). The main drivers of dead wood decay in aerated environments are commonly fungal community members, and are therefore key-players for such ecosystem functions (Hiscox et al., 2016; Leonhardt et al., 2019). A reduction in richness and diversity caused in many ecological processes a loss of functional ecological services (Singh et al., 2014; Jung et al., 2016), which were accompanied by a reduced ecological resilience (Griffiths et al., 2000).

Bacterial and fungal co-occurrence patterns differed strongly between natural and richness-reduced diversity (Figures 4, 5). We assume that the co-occurrence patterns of the natural diversity were less complex and governed by co-presence patterns (Figure 5A), reflecting an established community with structured functioning patterns. In contrast, the patterns of the richness-reduced diversity were more complex and were controlled by mutual exclusion patterns with few clusters of co-presence, indicating a dynamic community with disturbed functioning patterns. Therefore, the richness-reduced diversity struggled to recover while the natural diversity were able to resist according to the definitions of Oliver et al. (2015), indicating that microbial interaction patterns of the natural diversity were already established to cope with a fluctuating temperature regime.

The OTU assignment to network roles pointed out, that the majority of connectors, module and network hubs were found in the richness-reduced diversity (Table 3 and Figure 4). OTUs which were assigned to connectors, had high connectivity among clusters while network hubs had high connectivity within their cluster and among clusters. Module hubs are highly specialized within their cluster and had less connectivity among clusters while peripherals had low connectivity within their cluster and among clusters (Olesen et al., 2007). The richness-reduced diversity included 20 connectors, two network hubs and three module hubs indicating that the reduction in OTU richness removed established organismic setups toward a reassembly of OTU co-occurrence patterns and their associated ecosystem functions. In contrast, the natural diversity had one connector and three module hubs, indicating a stabilized functional network. OTU abundance were less important for co-occurrence patterns (Supplementary Table S3), indicating that functional traits of each OTU were more relevant. Functional guilds, e.g., denitrifying bacteria, were found frequently as connectors, module hubs or network hubs in the richness-reduced community (Table 3), highlighting their relevance in the present functional traits. Likewise, *Micropruina* sp. (network hub, 142), *Stenotrophomonas* sp. (connector, 203), and *Opitutus* sp. (module hub, 205) were capable of nitrate reduction (Shintani et al., 2000; Heylen et al., 2007) and were of high importance of the functional ecological processes in the richness-reduced diversity network. In late stage of decay, dead wood released nitrogen (Laiho and Prescott, 2004) and dead wood leachates contained the most nitrate and nitrogen content, and therefore denitrification processes were relevant. Both, *Micropruina* sp. and *Stenotrophomonas* sp. showed few co-occurrences in the natural community, but both genera and *Opitutus* sp. showed a high number of mutual exclusions in the richness-reduced diversity. The loss of co-presences of these potential key nitrate reducers may contribute to a reduced wood decomposition rate in the richness-reduced diversity. To unravel complex microbial community structures and their ecological function a deeper insight of their functional benefit is of high interest. In addition, our laboratory based findings should be explored on field sites to broaden our understanding on ecosystem scale.

## Conclusion

Our results showed that a richness-reduced microbial diversity was affected by temperature regime, which in turn had an impact on both dead wood decomposition and wood pH. These findings indicate that a richness-reduced diversity was more sensitive to fluctuating temperatures and thereby less effective in wood decomposition compared to the natural diversity. In turn, the microbial community with natural diversity was able to cope better with fluctuating temperatures based on a higher OTU richness and evenness, which enabled stabilized ecosystem services. The co-occurrence patterns of both diversity levels reflect that microbial functional traits were of paramount importance to ecosystem functioning rather than only fungal or bacterial richness. Therefore, our results support the insurance hypotheses and complement the effect of temperature impact on a microbial functional level.

## Data Availability Statement

The 16S rRNA gene and ITS2 region sequences are publicly available and deposited at https://www.bexis.unijena.de/PublicData/PublicData.aspx and can be found in the data sets 26346, 26366, and 227 26426.

## Author Contributions

MN constructed the hypotheses and questioning. SM prepared the experimental design. FM and SM performed the laboratory work, while the gas analysis was carried out by SH and MAH. SM did the final data analysis. SM and MN completed the writing. MN and MAH performed review and editing. All authors contributed to the article and approved the submitted version.

## Conflict of Interest

The authors declare that the research was conducted in the absence of any commercial or financial relationships that could be construed as a potential conflict of interest.

## Footnotes

1 www.biodiversity-exploratories.de
