# Supplementary material for: Fungal and Bacterial Diversity Patterns of Two Diversity Levels Retrieved From a Late Decaying Fagus sylvatica Under Two Temperature Regimes
Source: Front Microbiol. 2021 Jan 11;11:548793. doi: 10.3389/fmicb.2020.548793 (PMC7874115; doi:10.3389/fmicb.2020.548793)
Supplement: Supplementary file 1 [file Data_Sheet_1.pdf]

Supplementary Tables and Figures to

Fungal and bacterial diversity patterns of two diversity levels retrieved from a late decaying *Fagus sylvatica* under two temperature regimes

Sarah Muszynski[1], Florian Maurer[1], Sina Henjes[2], Marcus A. Horn[2], Matthias Noll[1*]

[1]Institute of Bioanalysis, Department of Applied Science, University of Coburg, Coburg, Germany

[2]Institute of Microbiology, Leibniz University of Hannover, Hannover, Germany

[*]**Correspondence:**
Matthias Noll; matthias.noll@hs-coburg.de

Table S1: Taxonomic classification and relative sequence read abundances of the sterile diversity. **Separate excel file.**

Table S2: Temporal shifts in the topological role of OTUs in microbial networks derived from the natural or richness-reduced community over incubation time (weeks). All OTUs were categorized into network hubs, module hubs, connectors or peripherals according to Olesen et al. (2007) (see also Figure 3).

Incubation Time (weeks)	Peripherals	Connectors	Module Hub	Network Hub
Natural community				
1	6	0	0	0
2	27	0	2	0
3	20	2	0	0
4	52	0	0	0
5	50	2	2	0
6	62	3	2	1
7	118	3	5	0
8	127	1	3	0
Richness-reduced community				
1	71	1	3	0
2	-	-	-	-
3	111	2	1	0
4	43	17	0	1
5	149	19	3	2
6	134	31	0	5
7	-	-	-	-
8	177	20	3	2

-: Calculation of network and topological role was not possible

Table S3: Taxonomic classification and functional traits of topological noticeable OTUs in a microbial network derived from the natural (N) and richness-reduced (R) diversity after 8 weeks of incubation. OTUs of the network assigned to the connectors, module hubs, network hubs or peripherals suggested by Olesen et al. (2007) were listed (see also Figure 3).

Separate excel file.

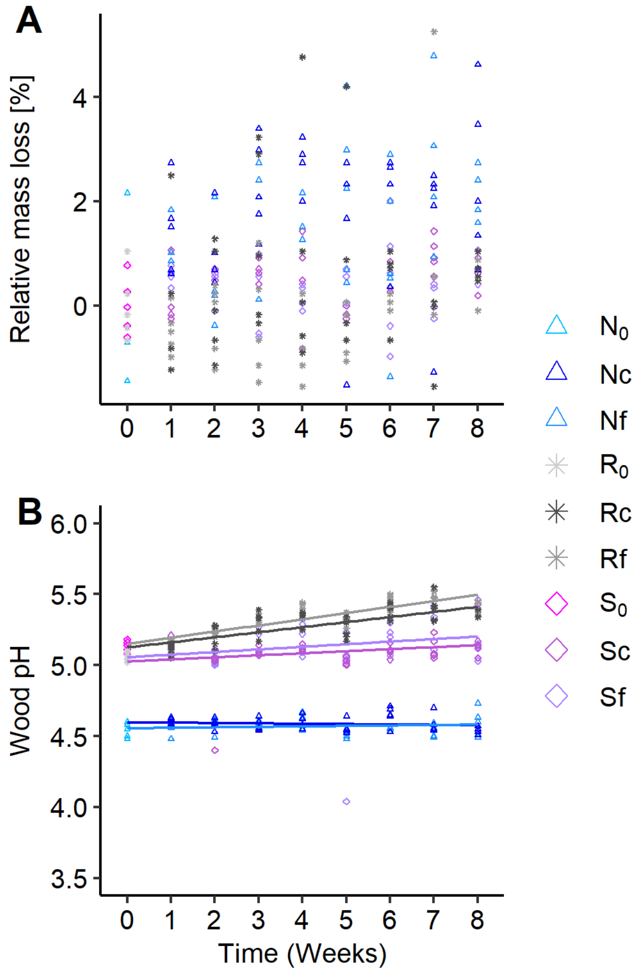

	R^2	*p*-value
Relative mass loss		
Nc	0.2	0.24
Nf	0.31	0.088
Rc	0.078	0.65
Rf	0.28	0.099
Sc	0.22	0.22
Sf	0.13	0.43
Wood pH		
Nc	-0.22	0.16
Nf	0.07	0.67
Rc	0.69	**<0.0001**
Rf	0.81	**<0.0001**
Sc	0.13	0.43
Sf	0.29	0.065

Figure S1: Relative wood mass loss (A) and wood pH (B) from *Fagus sylvatica* dead wood chips under two temperature regimes (fluctuating, f and constant, c) and three diversity levels (natural, N, richness-reduced, R and sterile, S) over 8 weeks of incubation. Relative wood mass loss was defined as percentage change from the initial wood mass (N_0, R_0 and S_0). Correlation coefficient and associated p value ($p \leq 0.05$) were calculated for each diversity level (N, R, S) and temperature regime (c, f), and were indicated by a linear regression (see table to the figure). See legend for symbol of each diversity level and temperature regime.

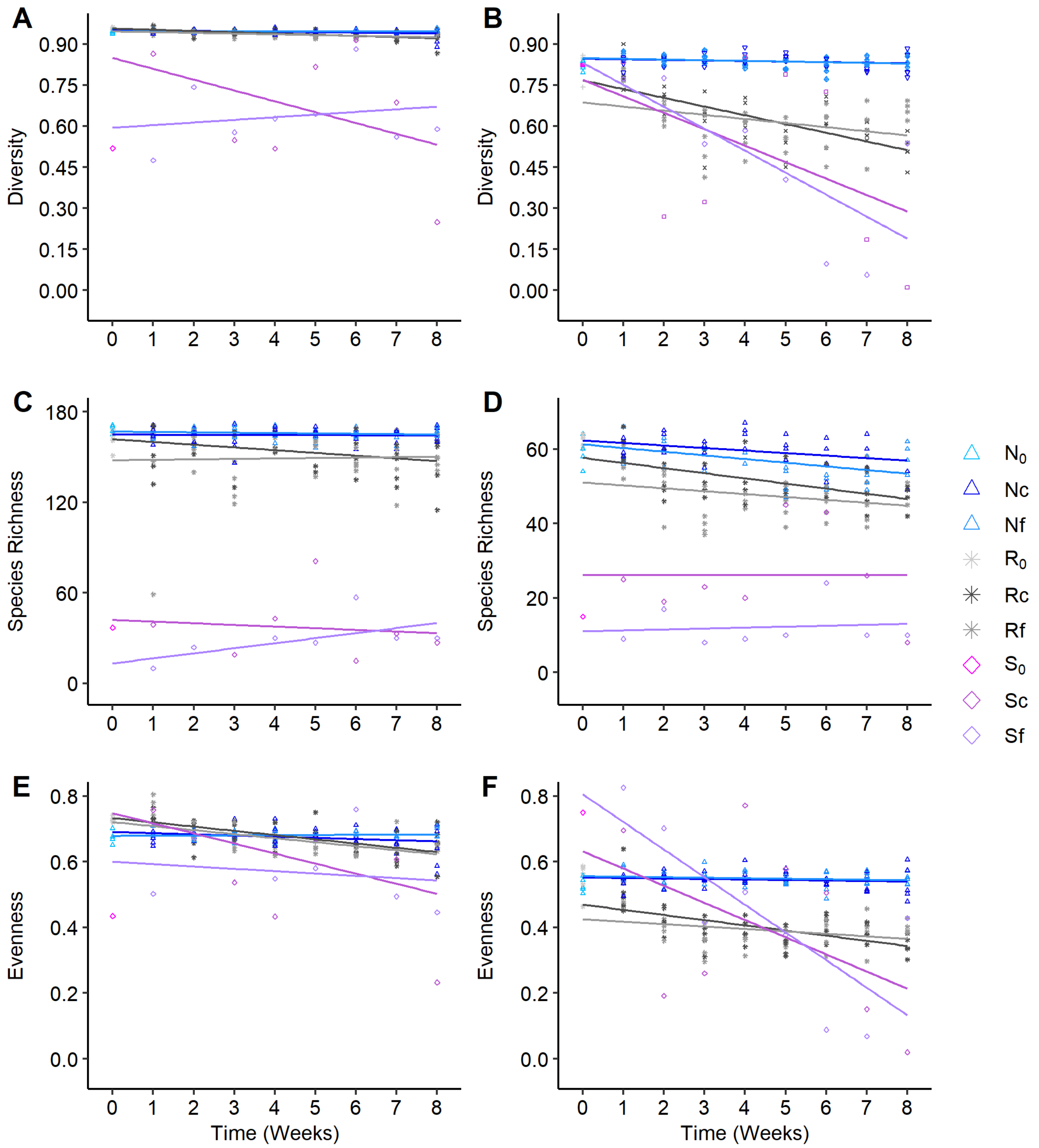

Figure S2: Simpson`s diversity, species richness and evenness for bacterial (A, C, E) and fungal (B, D, F) community of *Fagus sylvatica* dead wood chips under two temperature regimes (fluctuating, f and constant, c) and three diversity levels (natural, N, richness-reduced, R and sterile, S) over 8 weeks of incubation. Correlation coefficient and associated p value ($p \leq 0.05$) were calculated for each diversity level (N, R, S) and temperature regime (f, c), and were indicated by a linear regression (see table to the figure). See legend of figure S1 for symbol of each diversity level and temperature regime.

		R^2	*p*-value		R^2	*p*-value
Bacteria				***Fungi***		
Simpson Diversity	Nc	-0.073	0.66		-0.12	0.45
	Nf	-0.0089	0.96		-0.21	0.21
	Rc	-0.54	**<0.001**		-0.74	**<0.0001**
	Rf	-0.51	**<0.001**		-0.27	0.087
	Sc	-0.29	0.56		-0.55	0.17
	Sf	0.14	0.75		-0.087	0.069
Species Richness	Nc	-0.072	0.66		-0.33	**0.04**
	Nf	-0.17	0.31		-0.48	**0.002**
	Rc	-0.31	0.056		-0.51	**<0.001**
	Rf	-0.18	0.27		-0.3	0.059
	Sc	-0.25	0.59		0.071	0.88
	Sf	0.81	**0.016**		0.38	0.35
Evenness	Nc	-0.077	0.64		-0.15	0.37
	Nf	0.071	0.67		-0.13	0.43
	Rc	-0.6	**<0.0001**		-0.56	**<0.001**
	Rf	-0.64	**<0.0001**		-0.2	0.22
	Sc	-0.29	0.56		-0.55	0.17
	Sf	-0.31	0.46		-0.71	0.058

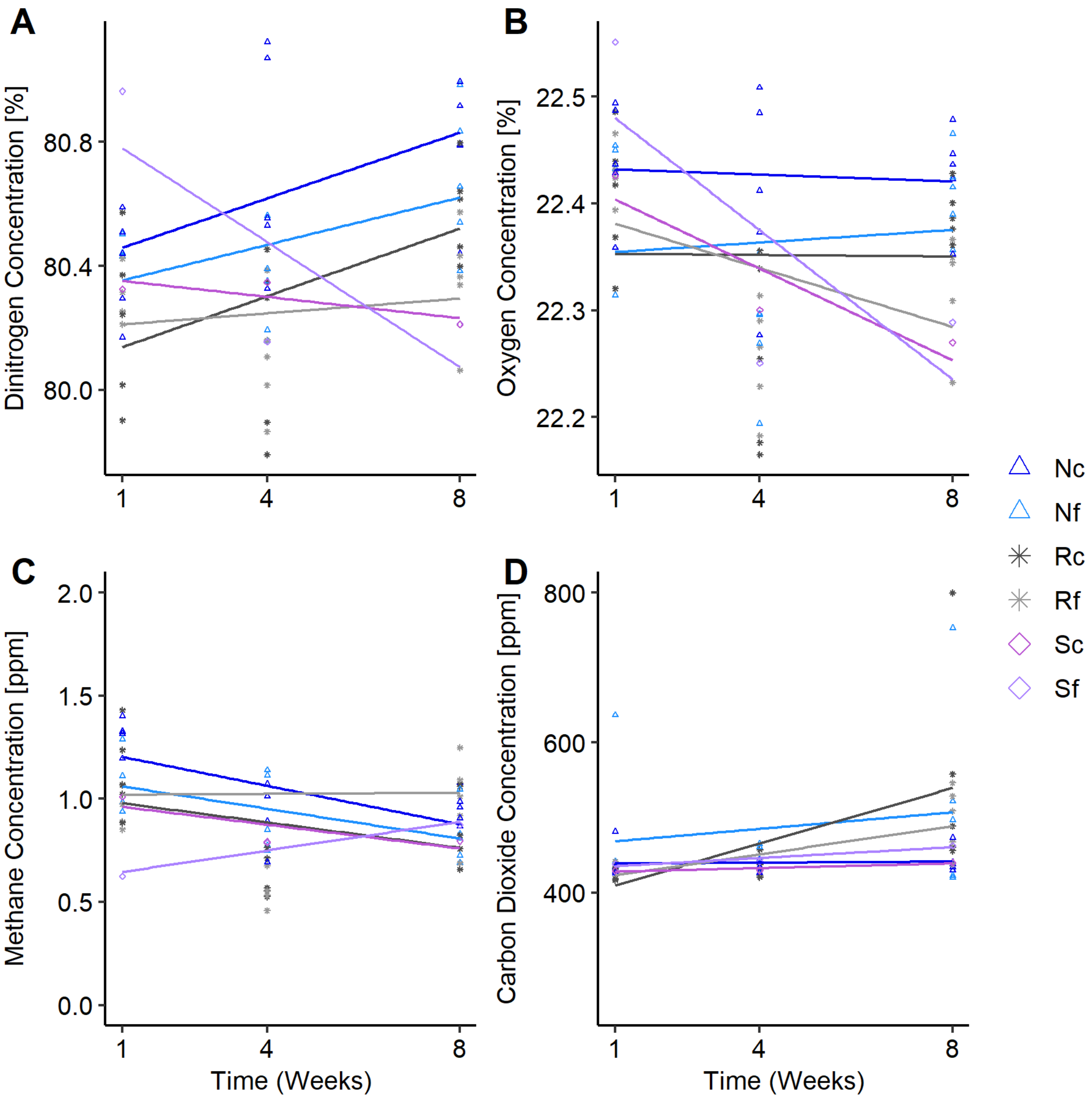

Figure S3: Concentration of dinitrogen (A) oxygen (B) methane (C) and carbon dioxide (D) in the headspace of *Fagus sylvatica* dead wood chips under two temperature regimes (fluctuating, f and constant, c) and three diversity levels (natural, N, richness-reduced, R and sterile, S) after one, four and eight weeks of incubation. For each diversity level (N, R, S) and each temperature regime (f, c) the correlation coefficient and the associated p value ($p \leq 0.05$) were calculated and indicated by a linear regression line. See legend of figure S1 for symbol of each diversity level and temperature regime.

	R^2	*p*-value		R^2	*p*-value
Dinitrogen			***Oxygen***		
Nc	0.53	**0.042**		-0.17	0.54
Nf	0.36	0.20		0.014	0.96
Rc	0.57	**0.028**		-0.057	0.84
Rf	0.19	0.5		-0.55	**0.034**
Sc	-0.50	1		-1	0.33
Sf	-0.50	1		-1	0.1
Methane			***Carbon Dioxide***		
Nc	-0.64	**0.0098**		0.23	0.42
Nf	-0.57	**0.035**		0.059	0.85
Rc	-0.34	0.21		0.77	**0.00069**
Rf	0.094	0.74		0.53	**0.043**
Sc	-0.50	1		1	0.33
Sf	1	0.33		1	0.33

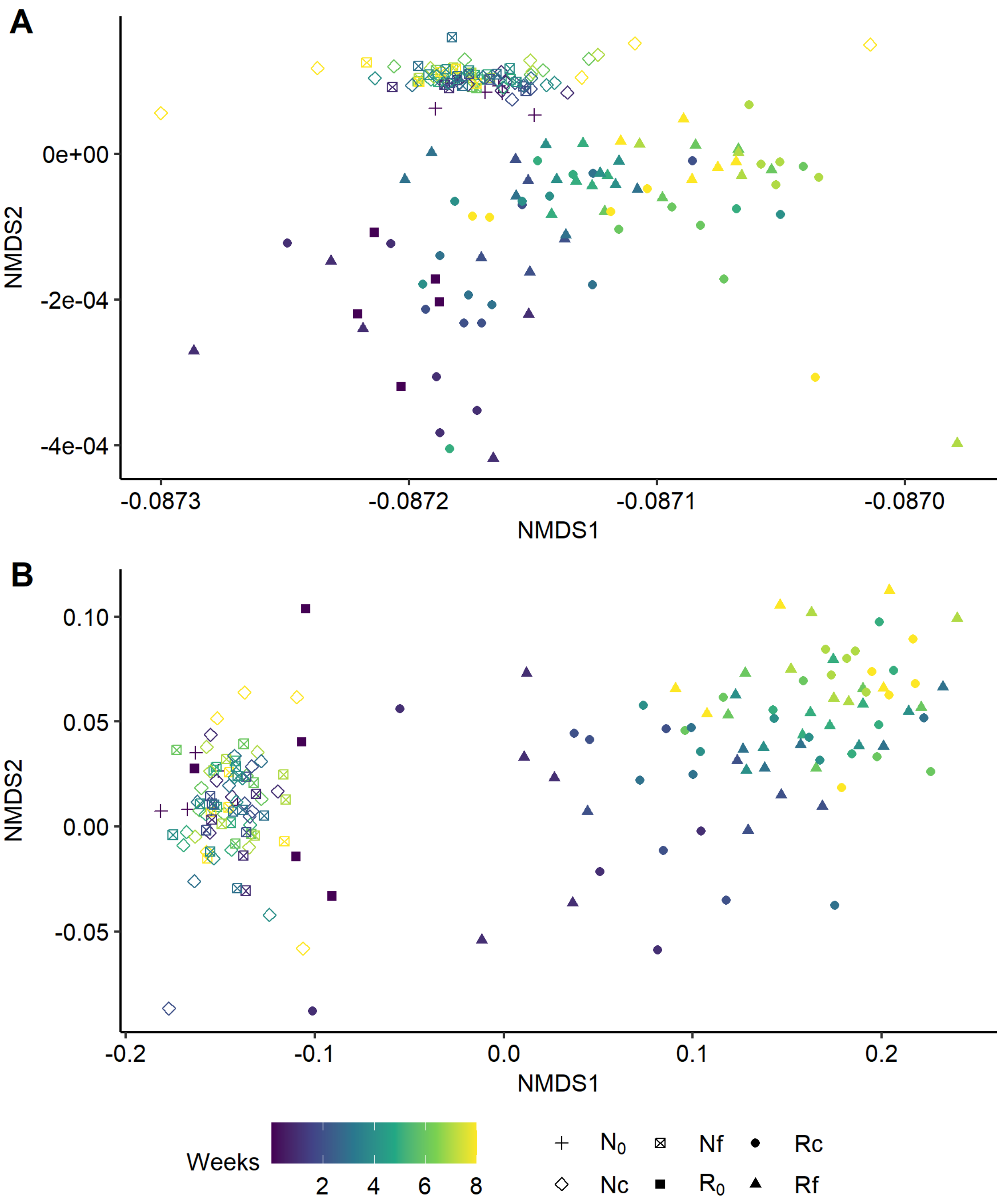

Figure S4: Non-metric multidimensional scaling (NMDS) of the bacterial (A) and fungal community composition (B) incubated in two temperature regimes (fluctuating, f ▲; and constant, c ●) and two diversity levels (richness-reduced, R; and natural, N). Diversity was calculated of the relative OTU abundances of after each week over an incubation period of eight weeks. Initial bacterial or fungal community composition is indicated as 0 (N_0 or R_0). NMDS is based on a Bray-Curtis dissimilarity matrix.
